# A deep learning model based on concatenation approach to predict the time to extract a mandibular third molar tooth

**DOI:** 10.1186/s12903-022-02614-3

**Published:** 2022-12-07

**Authors:** Dohyun Kwon, Jaemyung Ahn, Chang-Soo Kim, Dong ohk Kang, Jun-Young Paeng

**Affiliations:** Department of Oral and Maxillofacial Surgery, Samsung Medical Center, Sungkyunkwan University School of Medicine, 50 Irwon-Dong, Gangnam-Gu, Seoul, Republic of Korea

**Keywords:** Mandibular third molar, Extraction time, Predictive model, Concatenation approach, Artificial intelligence

## Abstract

**Background:**

Assessing the time required for tooth extraction is the most important factor to consider before surgeries. The purpose of this study was to create a practical predictive model for assessing the time to extract the mandibular third molar tooth using deep learning. The accuracy of the model was evaluated by comparing the extraction time predicted by deep learning with the actual time required for extraction.

**Methods:**

A total of 724 panoramic X-ray images and clinical data were used for artificial intelligence (AI) prediction of extraction time. Clinical data such as age, sex, maximum mouth opening, body weight, height, the time from the start of incision to the start of suture, and surgeon’s experience were recorded. Data augmentation and weight balancing were used to improve learning abilities of AI models. Extraction time predicted by the concatenated AI model was compared with the actual extraction time.

**Results:**

The final combined model (CNN + MLP) model achieved an R value of 0.8315, an R-squared value of 0.6839, a *p*-value of less than 0.0001, and a mean absolute error (MAE) of 2.95 min with the test dataset.

**Conclusions:**

Our proposed model for predicting time to extract the mandibular third molar tooth performs well with a high accuracy in clinical practice.

## Background

Extracting impacted mandibular third molar tooth is one of the most routine surgeries performed by oral and maxillofacial surgeons. Predicting the difficulty and time of tooth extraction is the most important factor to consider before performing a surgery [1]. In most of the previous studies on difficulty of extracting third molar tooth, tooth extraction difficulty assessment was performed based on radiological characteristics using the anatomical position, angulation, and adjacent anatomical structure of the mandibular third molar. MacGregor was the first to develop a model to predict operative difficulty using radiographs [2]. Pell & Gregory, Winter's classification and Pederson index are classification methods mainly used to predict the difficulty of third molar extraction. Based on these studies, many comparative studies and additional suggestions have been made [3, 4].

However, in many cases, the classification method does not match well with the actual clinical situation. Recently, a study using a convolutional neural network (CNN) to predict the difficulty of extraction of third molars from radiographic characteristics was published [5]. However, it had a limitation in that only radiological characteristics were considered when predicting the difficulty of tooth extraction using deep learning. Using anatomical elements of panoramic X-rays and clinical data as variables in a study using deep learning, it is possible to develop a better predictive model for the difficulty of extraction of the mandibular third molar. The time taken for the tooth extraction is one of the variables highly related to the surgical difficulty of the tooth extraction, and many dentists expect the expected extraction time before surgery [6]. Thus, in the present deep learning study, as a one way to predict the surgical difficulty of the mandibular third molar tooth extraction, the time taken for the extraction was predicted by considering both radiological and clinical factors such as gender, age, body mass index (BMI), and surgeon’s skill. Furthermore, the actual time taken for tooth extraction was compared with the time estimated through a deep learning model. The purpose of this study was to create a practical model for predicting the time to extract the mandibular third molar tooth using deep learning based on a concatenation approach.

## Methods

### Patients

In this study, panoramic images and clinical data from 724 patients aged 15 to 90 years who visited the Department of Oral and Maxillofacial Surgery, Samsung Medical Center from March 2020 to September 2020 were collected. Inclusion criteria were: (1) patient age between 15 and 90 years; (2) no relevant systemic diseases (American Society of Anesthesiologist’s classification ASA I and ASA II); and (3) no congenital and acquired deformity in the craniomaxillofacial area. Exclusion criteria were: (1) patients with systemic diseases (≥ ASA III); and (2) congenital and acquired deformity in the craniomaxillofacial area. Patients who were judged to require general anesthesia or sedation by the clinician were also excluded. The Institutional Review Board (IRB) of Samsung Medical Center approved this study (IRB number: 2021-11-109). All patients signed an informed consent agreement.

### Surgical technique

Extraction of mandibular third molars was performed by four operators (JYP, CSK, JMA, MKY) with 30, 24, 10 and 2 years of professional experience, respectively, as oral and maxillofacial surgeons using similar surgical techniques with the same instruments, high-speed and low-speed drills. Local anesthesia was administered with epinephrine 1:100.000 (Shinhung, Seoul, Korea) for the inferior alveolar nerve block and the third molar area around the gingiva. A 4–0 Vicryl suture (Ethicon Inc., Somerville, NJ, USA) was used to close the wound.

### Study variables

In this study, operation time was used as a target variable to measure the difficulty of extraction of the mandibular third molar. A set of predictor variables was divided into three groups: patient, operator, and radiologic variables. Patient variables included age, gender, BMI, and maximum mouth opening. Radiologic variables included the angulation, depth, bone density, morphology of the third molar, and the space between mandibular ramus and mandibular second molar. Operator variables included years of experience as an oral and maxillofacial surgeon and the class of each operator. Patient’s age, sex, body weight, height, and maximum mouth opening were recorded prior to extraction surgery. The time (minutes) from the start of the incision to the start of suture was recorded. Patient's body mass index (BMI) was calculated from their weight and height.

### Dataset

**Fig. 1:**
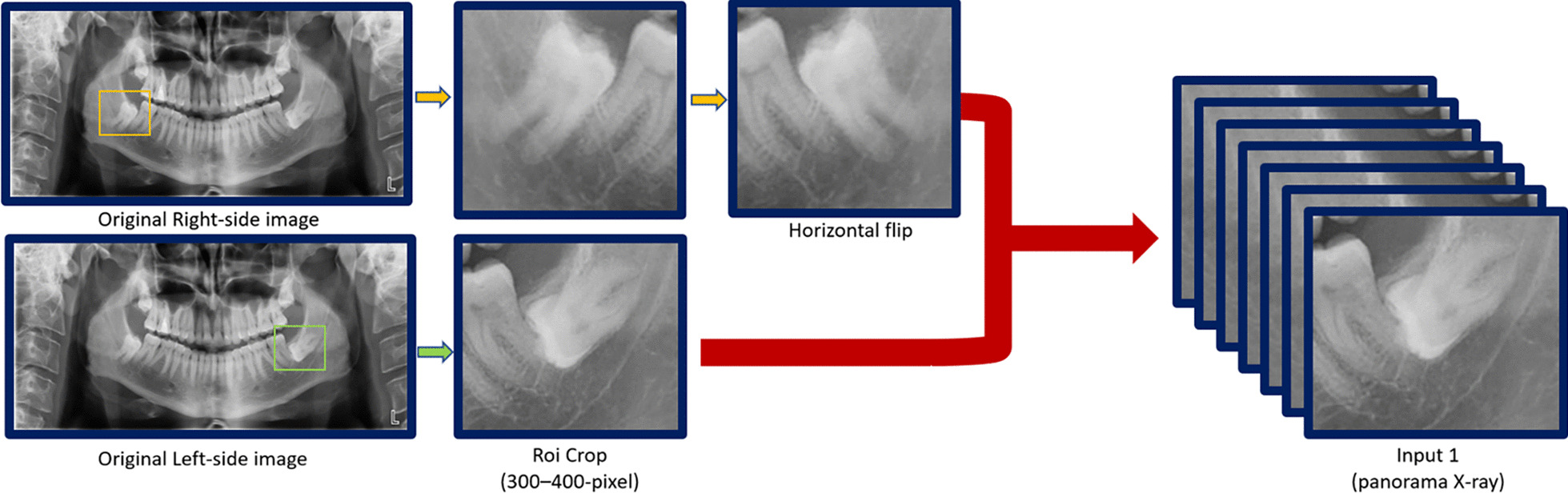
Processing of obtaining panoramic images

The region of interest (ROI) around the mandibular 3rd molar was manually cropped into a 300–400-pixel square shape with surrounding structures including the ramus of the mandible, distal part of mandibular 2nd molar, and inferior alveolar nerve. Consistency of ROI cropping was achieved in a way that two oral and maxillofacial surgeons with 10 and 25 years of experience reached consensus through discussion. The image of the right third molar was transformed into the image of the left through horizontal flipping to facilitate the deep learning process (Fig. 1). A total of 724 data were randomly classified into 644 training data and 80 test data set.

To test the accuracy of the AI model, validation was performed using data from additional 60 patients.

There was a case of data exclusion that the panoramic X-ray showed extra supernumerary tooth hiding behind the wisdom tooth.

### Deep learning model

**Fig. 2:**
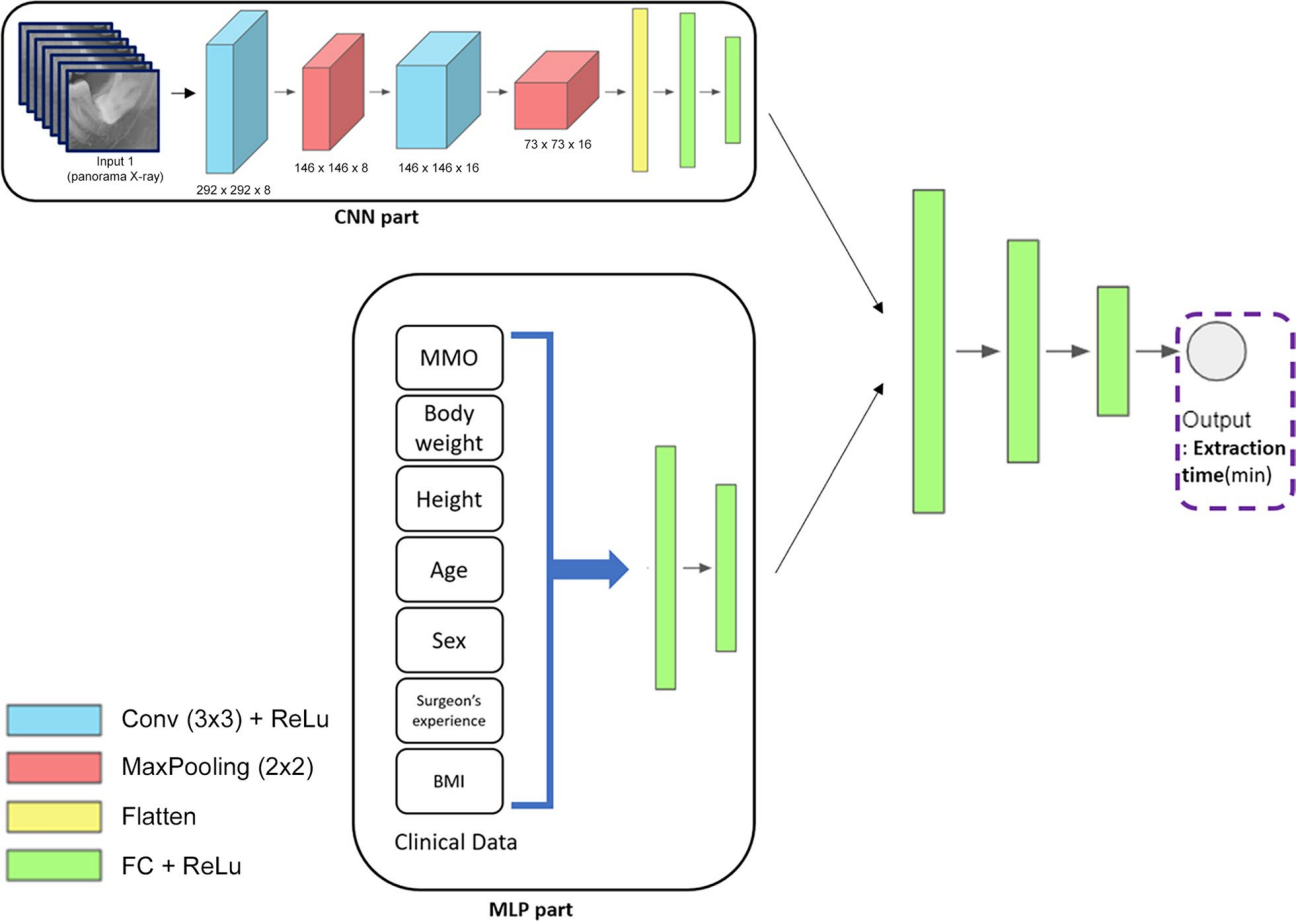
Overview of the concatenated model consisting of CNN part and MLP part. The CNN part learns image features through Convolutional Neural Network. The MLP part learns patients' clinical data through fully connected layers. Outputs from the MLP part and the CNN part are concatenated. Finally, there is an output layer that infers the extraction time, which is a positive integer, through fully connected layers. *CNN* Convolutional Neural Network; *MLP* Multilayer Perceptron

The deep learning model was constructed by concatenating Multilayer Perceptron (MLP) and Convolutional Neural Network (CNN) to handle two input data, panoramic X-ray images and clinical data. Figure 2 shows a clear description of the model which consists of the CNN part and the MLP part. The CNN part learns image features through Convolutional Neural Network with 3 × 3 filters, Max-pooling, Flatten, and Fully Connected layers. From the other side, the MLP part learns patients’ clinical data through fully connected layers. Outputs from the MLP part and the CNN part were concatenated. Finally, there is an output layer that infers the extraction time, which is a positive integer, through fully connected layers.

Rectified Linear Unit (ReLU) was used as the activation function. This has the advantage of quickly training the model and solving the vanishing gradient problem [7]. L2 Regularization is one of the weight decay methods that can train weights of the model to be not too big [8]. Although training data were increased through data augmentation, concerns about overfitting remained. Methods such as Ridge Regulation (L2 Regularization), Dropout, Early Stopping, and Batch Normalization were applied to the model for normalization. The Adagrad optimizer with default Keras settings for 200 epochs was found to work well for convergence.

### Grad-CAM

**Fig. 3:**
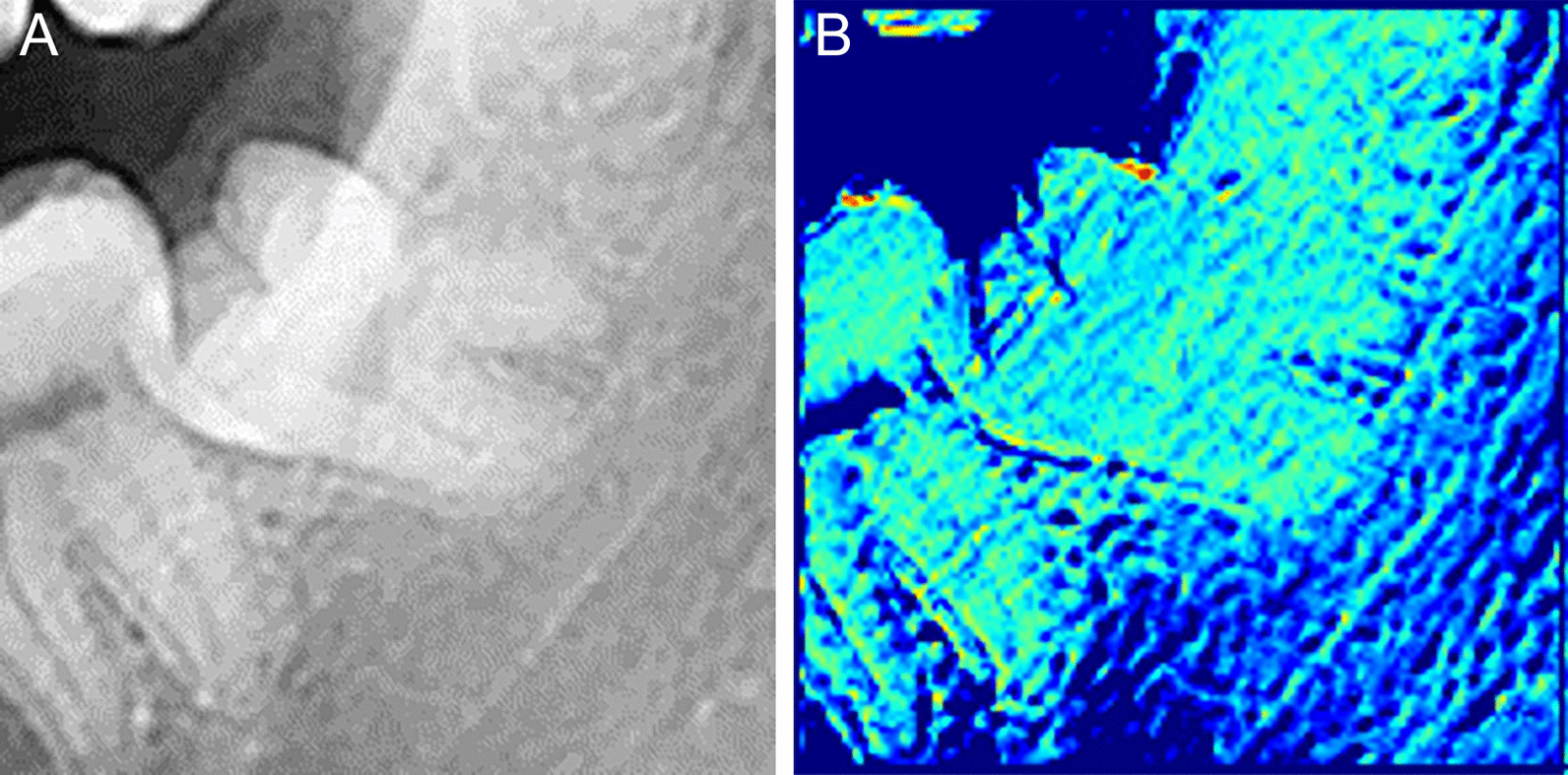
**a**, **b** Gradient-weighted Class Activation Mapping (Grad-CAM) was used to visualize which part of the panorama X-ray was referenced by the CNN model of this study when making a prediction

**Fig. 4:**
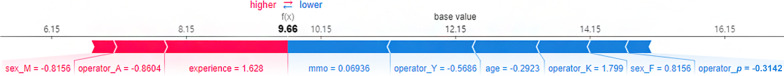
With SHAP (Shapley Additive exPlanations), which calculates the contribution of each feature of clinical data, we could visualize effects of each feature’s value in predictions made by the MLP

Deep learning models based on valid algorithms shows great performance. Sometimes it seems like a clear mathematical function. However, there is an opaque and complex process between its input and output. To solve this uncertainty, Gradient-weighted Class Activation Mapping (Grad-CAM) was used to visualize which part of the panorama X-ray was referenced by the CNN model of this study when making a prediction (Figs. 3a, b). With Shapley Additive exPlanations (SHAP) for calculating the contribution of each feature of clinical data, we could visualize effects of each feature's value in predictions made by the MLP (Fig. 4).

### Data augmentation

Data augmentation was applied to both images and clinical data classified as training sets. Changes applied to data augmentation included rotation (randomly within 10 degrees), horizontal shifting (horizontal shifting range = [− 20,20]), vertical shifting (vertical shifting range = 0.05), adjusting brightness (brightness range = [0.8,1,2]). No vertical or horizontal flipping was performed to prevent topographical changes in the left third molar.. In addition, augmentation was applied to clinical data matching each image by randomly changing values in a certain range (0 to 10% of each feature’s mean). As a result, the size of training dataset was increased from 644 to 8602.

## Results

### Distribution of patient, dental, and surgical variables

**Fig. 5:**
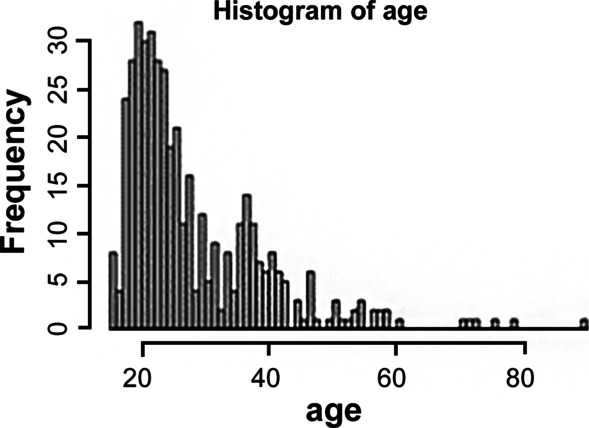
Age distribution of patients included in this study. Due to characteristics of wisdom tooth extraction, the proportion of young patients was high

A total of 724 patients were included in the study with a mean age of 28.6 ± 11.2 years (range 15–90 years) (Fig. 5). There were 355 men and 369 women. The mean body mass index (BMI) of patients was 22.8 ± 3.66. The average time taken for a third molar extraction was 12.9 ± 7.7 min (range 1–50 min). The number of lower third molars was almost evenly distributed (right: 351; left: 373). A simple comparison of extraction time according to gender (*p* = 0.24) and sides (*p* = 0.17) showed no statistically significant difference. The correlation between the maximum amount of mouth opening and extraction time was found to be statistically significant (Pearson’s correlation r = − 0.2, *p*-value < 0.001). The smaller the maximal opening, the more time it took for tooth extraction. The extraction time was further increased as patient’s age exceeded 50 years (*p* < 0.05).

**Fig. 6:**
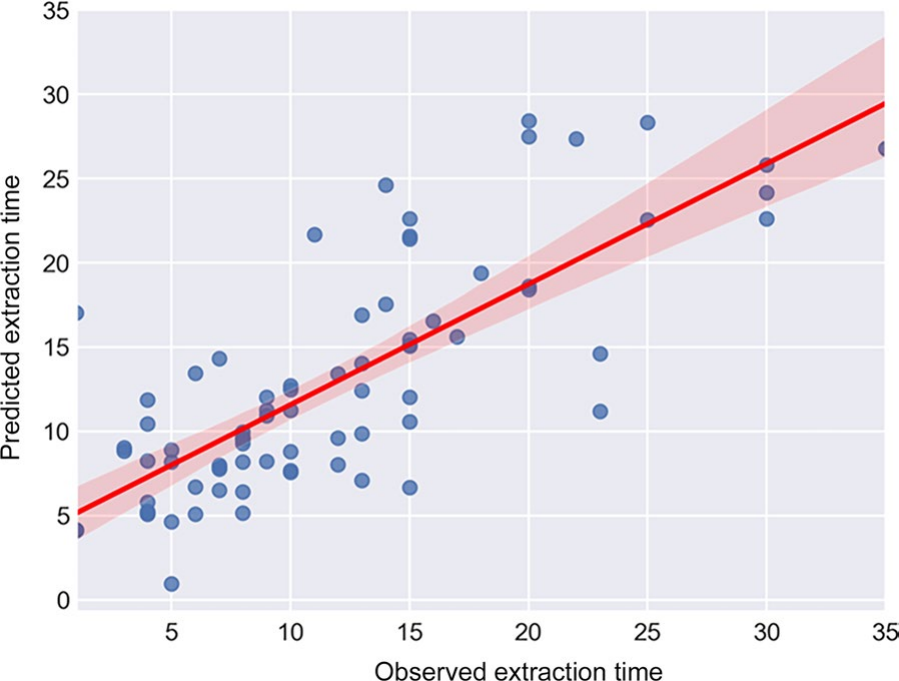
Correlation coefficient was 0.8315 (*p*-value < 0.05). The paired t-test between two groups showed a *p*-value more than 0.05. The mean absolute error (MAE) was 2.95 min

To evaluate the prediction model, the predicted extraction time and the actual time spent on extraction were compared through correlation analysis. The correlation coefficient was 0.8315 (*p*-value < 0.05). The paired t-test between two groups showed a *p*-value of more than 0.05. The mean absolute error (MAE) was 2.95 min (Fig. 6).

### Performance evaluation

Table 1 shows performance metrics of CNN, MLP, and Combined Model (CNN + MLP). The combined model was better in all measures of R-value, R-squared, *p-*value, and Mean Absolute Error (MAE) than when CNN and MLP were used separately. When CNN and MLP were used alone, extraction time was predicted only based on images or clinical data, respectively. However, in clinical practice, both panoramic images and clinical data are required to properly predict the extraction time. This means that models trained with both images and clinical data are expected to show better performance than using CNNs and MLPs separately.

**Table 1:** Metrics used for performance evaluation

	MLP	CNN	MLP + CNN
R-value	0.7000	0.3877	0.8315
R-squared	0.4696	0.1103	0.6839
*p*-value	1.3994e^−12^	0.0005	7.8256e^−21^
MAE(min)	4.1213	5.3923	2.9548

*MLP*, Multilayer perceptron; *CNN*, Convolutional neural network; *MAE*, Mean absolute error

When 60 external validations were performed, the mean absolute error (MAE) was 4.66 min and standard deviation was ± 3.72 min.

## Discussion

Extracting the impacted mandibular third molar is one of the routine procedures performed by oral and maxillofacial surgeons [1]. However, the difficulty of extraction varies from simple extraction to cases requiring general anesthesia. Categorizing the difficulty of extractions or estimating the time required has been of interest to oral and maxillofacial surgeons.

MacGregor made the first attempt to establish a model for assessing surgical difficulty [2]. Winter's and Pell & Gregory classification is a classic method [2, 9, 10]. Many studies have found that radiological factors are related to surgical difficulty for both upper and lower third molars, with depth of impaction, distal space available, molar angulation, and root morphology being main variables contributing to the difficulty [11–14]. However, these methods have recently been found to be inappropriate for judging the difficulty of surgery. The Pederson index showed low sensitivity and specificity in predicting the difficulty of surgery for impacted mandibular third molars [15]. Furthermore, the unreliability of radiographs for classification of impacted third molar irrespective of training or experience of the evaluator has been reported [15–17]. Other important variables not calculated by Pederson include maximum mouth opening, age, bone density, body mass index (BMI, kg/m^2^), and proficiency of the surgeon.

Most of the previous deep learning approaches related to the difficulty prediction of third molars used panoramic radiographs only. De Tobel et al. [18] developed an automated method to assess the degree of development using mandibular third molars on panoramic radiographs. Another study that predicted the difficulty of wisdom tooth extraction using CNN considered only radiological characteristics for the prediction [5] Furthermore, most of the previous AI-based difficulty prediction study were difficulty classifiers or wisdom tooth detectors rather than predicting the actual wisdom tooth extraction time[5]. Other study used AI-driven molar -angulation measurement and predicted the third molar eruption potential [19]. Zhu et al. [20] developed a deep learning based-detection model for assessing the contact relationship between wisdom tooth and the inferior alveolar nerve based on panoramic X-rays.

Unlike other deep learning studies using only panoramic X-ray or cone beam computed tomography (CBCT), this study is significant in that it is the first study to create a practical model for predicting the time to extract the mandibular third molar by considering both panoramic X-ray and clinical data. Our proposed deep learning model predicts the time taken for extraction like clinicians predicts the estimated time considering both radiographic information and clinical information. A statistically significant positive correlation between the predicted extraction time and real extraction time was observed.

In this study, a set of predictor variables were divided into three groups: patient, operator, and radiologic variables. Patient variables included age, gender, BMI, and maximum mouth opening. Operator variable included the surgeon’s years of experience based on the years of practice as an oral and maxillofacial surgeon and the class of each operator. Radiologic variables included angulation, depth, bone density, morphology of the third molar, and the space between mandibular ramus and mandibular second molar. These radiological factors were not specified as numerical or nominal variables. Radiographs were imported as image data and included into the CNN model.

Shapley Additive exPlanations (SHAP) for calculating the contribution of each feature of clinical data was used in this study. The goal of SHAP is to account for the prediction of an instance by calculating the contribution of each feature to the prediction. The Python SHAP package allows you to visualize feature attribution as "forces" as Shapley values [21]. In this AI model, features with the largest absolute Shapley value was operator's clinical experience(years). Followed by the maximum mouth opening (MMO) (Fig. 5) In Table 1, when CNN and MLP were used alone, MAE(min) was 4.1213 and 5.3923 respectively. This result tells us that MLP showed better prediction than CNN. The combined model was even better in all measures than when CNN and MLP were used separately. However, it was difficult to clearly demonstrate how much each factor affects in the CNN + MLP combined model. When looking at the SHAP result in the MLP model, it can be interpreted that the operator's clinical experience(years) had a great influence. Although these predictors used in this study have not been used in other AI-based predictive studies of wisdom tooth extraction, most clinicians understand that these variables are essential for predicting extraction time and difficulty.

Methods such as Ridge Regulation (L2 Regularization), Dropout, Early Stopping, and Batch Normalization were applied to the model for normalization. Dropout can prevent the model from overfitting in a way that does not involve some weights in training. Early Stopping is a method of terminating training if the performance of the model for the validation set does not improve any more during the epoch. Batch Normalization is a way to train a model faster and more stably by normalizing the input distribution of each layer. Data augmentation is commonly used to overcome limitations of small data sets that are unique to the medical field. The size of each training dataset was increased from 624 to 8602 after data augmentation.

Renton has reported that age, patient weight, and ethnicity are associated with extraction times [3]. When patients were divided by age, those over 30 years were at a significantly more risk of difficult extractions than younger patients. The difficulty was further increased as patient's age exceeded 50 years [3]. However, in the present study, no significant differences in the difficulty of wisdom tooth extraction were found according to age. This might be because age distribution of the study population was biased towards younger patients. A majority of patients included in the study were in their 20 s (Fig. 5).

To the best of our knowledge, this is the first study to predict the time to extract the wisdom tooth through artificial intelligence using both panoramic images and clinical data. This study has some limitations. First, the study population of the study was skewed toward the younger age group. The number of subjects in the age group of more than 50 years in the study population was limited. Such age inhomogeneity of this study group might have led to less consideration of the age factor when artificial intelligence was used to predict the difficulty of tooth extraction. Most of other variables showed normal distribution in this study. Although the age distribution of the patients was skewed, no further action was taken as our AI model was already showing good results. If the number of subjects in the entire study group is increased through additional studies in the future with age group of patients uniformly included, a more sophisticated extraction difficulty prediction model is expected to emerge.

In addition, in this study, a panoramic image was used as an image variable. If CBCT data are used for the same prediction model in the future, a more sophisticated model might be obtained.

Experienced dentists and oral and maxillofacial surgeons predict and prepare for the difficulty of tooth extraction by considering various factors such as panorama, CBCT, patient's age, gender, and morphology. However, in the case of novice dentists, the difficulty is often unpredictable. Novice dentists often fail to predict the difficulty of extraction, leading to a very long extraction time or an increase in patient discomfort after surgery. There is no clinician who judges the difficulty of extraction using only panoramic X-ray or cone beam CT images, but comprehensively predicts through various information such as the patient's age, BMI, morphology, and gender. The AI prediction model of this study is an AI model that can predict the time to extract the wisdom tooth by considering both radiographic images and patient's clinical data rather than simply predicting the time through AI using a panoramic image. This can be of great help to novice dentists when predicting the difficulty and time before wisdom tooth extraction or when deciding whether to refer to a specialist without extraction.

## Conclusions

We proposed a concatenated model combining a convolutional neural network (CNN) model using an X-ray image (panoramic view) and a multilayer perceptron (MLP) model using patient's clinical data to predict the time to extract a mandibular third molar tooth. This concatenated model accurately predicted the time to extract the third molar tooth in actual clinical practice.

## Data Availability

The datasets used and/or analyzed during the current study are available from the corresponding author on reasonable request.

## Abbreviations

AI: Artificial intelligence
CNN: Convolutional neural network
MLP: Multilayer Perceptron
MAE: Mean absolute error
BMI: Body mass index
ASA: American society of anesthesiologist’s classification
ROI: Region of interest
ReLU: Rectified linear unit
Grad-CAM: Gradient-weighted class activation mapping
SHAP: Shapley additive explanations
CBCT: Cone beam computed tomography
